# Geographic Origin and Functional Group Type Affect the Decomposability of Peatland Plant Litter Through Biochemical Properties

**DOI:** 10.1002/ece3.71758

**Published:** 2025-07-07

**Authors:** Jinze Ma, Yong‐Da Chen, Si‐Nan Wang, Jing Zeng, Chao Liu, Zhao‐Jun Bu

**Affiliations:** ^1^ School of Geography and Environmental Engineering Gannan Normal University Ganzhou China; ^2^ Jilin Provincial Key Laboratory for Wetland Ecological Processes and Environmental Change in the Changbai Mountains Institute for Peat and Mire Research Changchun China; ^3^ Key Laboratory of Geographical Processes and Ecological Security in Changbai Mountains, Ministry of Education, School of Geographical Sciences Northeast Normal University Changchun China; ^4^ Ecological and Landscape Engineering Technology Innovation Center of the Ministry of Housing and Urban Rural Development China Urban Construction Design & Research Institute Beijing China

**Keywords:** biochemical properties, climate warming, decomposition, functional group, latitude, *sphagnum*

## Abstract

Peatlands are the most important global soil carbon reservoirs due to low decomposition rates. However, the influence of peatland plant litter quality and geographic environment on decomposition remains poorly understood. This article aims to explore the interactive effects of geographical origin and plant functional groups on the initial chemical characteristics and decomposition processes of peatland litter, elucidating how climate‐driven biochemical legacies regulate decomposition dynamics. Plant materials were collected from three peatlands in East China spanning distinct bioclimatic regimes along a latitudinal gradient: Dajiuhu (31°29′N), Hani (42°13′N), and Mangui (52°19′N) peatlands. A 3‐year decomposition experiment was conducted in Hani peatland starting in October 2014, using standardized litter bags containing nine species from three functional groups: mosses (*Sphagnum*), graminoids (*Carex*), and shrubs (*Betula*). Fresh plant materials from all sites was translocated to Hani peatland for decomposition experiment. Decomposition rates and key biochemical traits were quantified through sequential destructive sampling at 0‐, 1‐, 2‐, and 3‐year post‐deployment. Results showed decomposition rates of plant litter descending as *Carex* > *Betula* > *Sphagnum*. Latitudinal origin significantly affected decomposition; the northernmost Mangui litter exhibited the lowest mean decomposition (41.1%) compared to Hani (45.4%) and Dajiuhu (44.4%). Total phenolics (β = −0.62) and lignin (including *Sphagnum*‐specific lignin‐like substances, β = −0.93) significantly inhibited decomposition. Interactive effects between geographical origin and functional group were significant, with *Carex* decomposition most sensitive to latitude. These findings demonstrate that geographic environment and plant functional group type jointly regulate peatland litter decomposability through biochemical traits. Climate warming may accelerate decomposability, potentially diminishing the carbon accumulation function of peatlands, underscoring the necessity to integrate latitudinal gradients and functional diversity into peatland carbon models.

## Graphical abstract

This study investigated the influence of geographical origin and plant functional groups on peatland litter decomposition. Results demonstrated that geographic environment and plant functional group type jointly regulate peatland litter decomposability through biochemical traits. Climate warming may accelerate decomposition, potentially diminishing peatlands' carbon sink function.

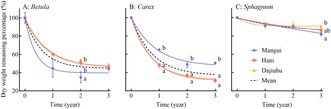

## 1 Introduction

Peatlands hold approximately one‐third of the global soil organic carbon (Dorrepaal et al. 2009; Yu et al. 2010); this substantial carbon storage is attributed to the slow decomposition of organic matter (Wang et al. 2021). In peatlands, waterlogged conditions and recalcitrant litter (e.g., *Sphagnum*) synergistically inhibit microbial activity, forming unique biochemical legacy effects that stabilizes carbon stocks (Fenner and Freeman 2020; Ofiti et al. 2023). The impact of climate change on litter decomposition in peatlands has attracted widespread attention; however, mechanisms linking geographic gradients, functional traits, and biochemical legacies remain underexplored in these ecosystems.

The quality of plant litter plays a crucial role in inhibiting decomposition within peatlands (Limpens et al. 2017). The decomposition rate of vascular plant leaf litter correlates with its initial lignin/N ratios in central Canadian peatlands (Moore and Basiliko 2006). In nutrient‐poor peatlands, plant litter contains lower nutrient levels compared to other ecosystems, further suppressing its decomposition (Aerts et al. 1999). *Sphagnum* litter exhibited a higher carbon to nitrogen ratio (C/N) and lower nutrient concentrations compared to vascular plants, thereby restricting decomposers' resource acquisition and decomposition efficiency (Zhang et al. 2022). Research on peatlands highlights that *Sphagnum* produces phenolic compounds and lignin‐like polymers, which are key drivers of recalcitrance (Wang et al. 2015; Kazakou et al. 2019). Additionally, lignin content in vascular plants regulates decomposition rates across peatland types (Liu, Zhang, et al. 2024). In tropical forest peat, for example, high lignin content inhibited decomposition, counteracting the accelerated breakdown typically driven by elevated temperatures (Dommain et al. 2015).

A close relationship exists between plant growth rates and the decomposition rates of derived litter, primarily mediated through litter substrate quality (Wu et al. 2025). In peatlands, fast‐growing plants typically produce readily decomposable litter characterized by low C/N ratios and reduced lignin content. In contrast, slow‐growing plants generate litter with elevated C/N ratios and higher lignin content, resulting in significantly slower decomposition (Moore and Basiliko 2006; Zhang et al. 2022). Environmental factors (e.g., temperature, precipitation) indirectly regulate litter quality by modulating plant growth strategies (Zhang et al. 2021). Consequently, plant growth rates exert legacy effects on litter decomposition through biochemical modifications of litter quality.

Geographic environmental factors influence decomposition rates at larger scales. Zhang et al. (2008) demonstrated that litter decomposition rates decrease with increasing latitude. In peatlands, latitudinal gradients correlate with shifts in temperature, hydrology, and vegetation composition, collectively altering litter chemistry and microbial activity (Dorrepaal et al. 2005; Joly et al. 2023). For instance, colder high‐latitude peatlands favor *Sphagnum* dominance and peat accumulation, while warmer regions exhibit higher vascular plant productivity and labile litter inputs (Dommain et al. 2015; Wang et al. 2015). Climate is the primary regulator of litter decomposition at broad geographic scales, with annual mean temperatures driving decomposition rates along latitudinal gradients (Ge et al. 2017; Zhang et al. 2021). Species‐driven effects on decomposition outweigh climatic controls at local scales (Djukic et al. 2018). Geographic environmental factors regulate litter decomposition through dual pathways: directly by altering decomposition processes (Van Zuijlen et al. 2020), and indirectly by reshaping plant community composition and phenology (Dorrepaal et al. 2005; Song et al. 2014), thereby modifying litter biochemical quality (Liu, Zhang, et al. 2024). Disentangling these direct and indirect effects is critical for elucidating mechanistic drivers of decomposition dynamics. However, the indirect effects of geographic environment on decomposition remain understudied (Joly et al. 2023; Wu et al. 2025).

Functional groups in ecology refer to species assemblages sharing analogous structural or functional traits, such as growth forms, life history strategies, or morphological adaptations (Thomas et al. 2019). Over evolutionary timescales, environmental adaptation has driven functional groups of plants to develop specific traits, including unique chemical compositions in litter, which critically influence litter decomposition dynamics (Yu et al. 2024). In peatlands, functional groups (e.g., mosses, graminoids, shrubs) exhibit distinct trade‐offs between nutrient acquisition and stress tolerance, shaping litter chemistry and decomposability (Moore and Basiliko 2006; Kazakou et al. 2019). For example, graminoids produce nitrogen‐rich litter with rapid decomposition, whereas *Sphagnum* form persistent litter due to high phenolic content (Limpens and Berendse 2003; Bragazza et al. 2009).

Functional groups also shape large‐scale biogeographic decomposition patterns. Körner (1989) documented pronounced latitudinal gradients in graminoid decomposition rates, absent in evergreen woody plants. However, nutrient‐poor peatland plant litter often obscures functional‐group differences in decomposition biogeography (Dorrepaal et al. 2005). Despite the ecological significance, comparative studies on litter decomposability across latitudinal gradients, particularly in peatlands, remain limited (Liu et al. 2018; Joly et al. 2023).

Plant litter decomposability is fundamentally governed by its biochemical quality. Plants from distinct geographic origins and functional groups exhibit divergent growth rates, leading to variations in litter quality and consequently decomposability. However, the interactive influence of geographical origin and plant functional groups on the initial chemical characteristics and decomposition processes of peatland litter remains lack empirical validation. To address this, we selected three peatlands along a latitudinal gradient in East China—spanning subtropical to cold‐temperate zones—to represent contrasting bioclimatic regimes. Dominant species from three functional groups were studied: mosses (*Sphagnum* spp.), graminoids (*Carex* spp.), and shrubs (*Betula* spp.), all of which are representative taxa in three peatland ecosystems. By translocating litter to a common decomposition site (Hani peatland), we disentangled the intrinsic decomposability of litter from localized environmental influences. Three hypotheses were formulated: (1) leaf litter decomposition follows a functional hierarchy, with graminoids decomposing faster than woody plants and much faster than mosses; (2) Lower‐latitude origins enhance decomposability, with warmer origins (lower latitudes) litter decomposing faster; and (3) there is an interaction between geographical origin and functional groups on litter decomposition, with differential responses across functional groups.

## 2 Materials and Methods

### 2.1 Experimental Site and Species

Three peatlands in East China along a latitudinal gradient were selected to simulate climatic and ecological variability: Dajiuhu peatland (31°29′ N, 109°59′ E) in the Daba Mountains of Hubei Province, Hani peatland (42°13′ N, 126°31′ E) in the Changbai Mountains of Jilin Province, and Mangui peatland (52°19′ N, 122°26′ E) in the Greater Khingan Range of Inner Mongolia. These peatlands span distinct bioclimatic regimes, from subtropical evergreen broad‐leaved forests to temperate coniferous and broad‐leaved mixed forests, and then to cold‐temperate coniferous forests. During the study period, the annual mean temperatures at the three locations were approximately 7.9°C, 2.0°C, and −1.1°C, respectively. The annual precipitation ranges were 1535–2100, 757–930, and 350–500 mm, respectively. All three peatlands are dominated by *Sphagnum* and have well‐developed peat moss layers. *Carex* is one of the most dominant herbaceous vegetation types in these peatlands, primarily distributed on *Sphagnum* moss hummocks. *Birch* is the dominant woody plant in the marginal areas of the three peatlands and is also commonly distributed in open regions of the peatland systems. The plant size of *Betula*, *Carex*, and *Sphagnum* increases with decreasing latitude. Hani peatland, situated in a coniferous‐broadleaf transition zone, was selected as the experimental site due to its intermediate climate and representative *Sphagnum* peatland ecology.

In this study, common species including mosses (*Sphagnum*), graminoids (*Carex*) and shrubs (*Betula*) were selected from the three peatlands. In total, nine plant species were included (Table 1). During August 2014 (peak growing season, pre‐senescence), *Sphagnum* shoots along with fresh leaves from *Carex* and *Betula* were collected to serve as decomposition substrates in subsequent experiments. After air‐drying and aging at room temperature, samples were further dried in ovens at 30°C until they reached a constant weight (about 48 h). Both *Sphagnum* and *Carex* were cut into approximately 2‐cm segments. For each species, 2.0 g of litter was weighed and placed into 90‐mesh nylon decomposition bags. This sample size was determined based on preliminary trials and previous research indicating that the remaining mass after 3‐year decomposition (typically 40%–95% of initial weight under local conditions) would provide sufficient material (≥ 0.7 g) for all chemical analyses (Moore and Basiliko 2006; Zhang et al. 2024). Bag dimensions were selected based on material volume: 10 cm × 10 cm for *Sphagnum* litter and 10 cm × 5 cm for vascular plant litter.

**TABLE 1:** The species used as litters in the experiment.

Functional group	Species	Origin
Shrub	*Betula albosinensis*	Dajiuhu
	*Betula ovalifolia*	Hani
	*Betula fruticosa*	Mangui
Graminoid	*Carex doniana*	Dajiuhu
	*Carex lasiocarpa*	Hani
	*Carex schmidtii*	Mangui
Moss	*Sphagnum palustre*	Dajiuhu
	*Sphagnum centrale*	Hani
	*Sphagnum magellanicum*	Mangui

### 2.2 Experimental Design and Measurement

In early October 2014, relatively flat *Sphagnum* hummocks in Hani peatland with a water table depth of 20–25 cm were selected as the experimental microsite. This water table range represents both the Hani peatland's long‐term average (Wang et al. 2025) and typical hydrologic conditions in boreal peatland ecosystems (Laiho 2006). Decomposition bags from three functional groups across three peatlands were randomly buried within these hummocks, positioned 4–6 cm below the hummock surface. Given the markedly slow decomposition rates under waterlogged/anoxic conditions in boreal peatlands, annual sampling intervals effectively captured incremental mass loss patterns, as implemented in comparable decomposition studies (Zhang et al. 2024). Decomposition bags were systematically retrieved in October immediately following burial and after 1‐, 2‐, and 3‐year intervals. In total, the experiment involved 180 decomposition bags (3 geographic origins × 3 functional groups × 4 decomposition times × 5 replicates). During retrieval, each decomposition bag was cleaned of surface impurities, sealed individually in zip‐lock bags, transported to the laboratory under refrigeration, and stored at −20°C in darkness until analysis. The 3‐year decomposition experiment was conducted from 2014 to 2017. While the original dataset was archived after completion, a recent reanalysis during ongoing research revealed novel insights into the latitudinal variation in peatland litter decomposability.

In the laboratory, roots and other impurities were removed from each decomposition bag. The plant litter was oven‐dried to a constant weight at 65°C. Initial and decomposed dry weights were measured using a balance (BSA223S, Sartorius, Germany) with a resolution of one‐thousandth of a gram. After the litter was ground into a fine powder, chemical properties were analyzed. Total carbon and total nitrogen content were determined by the combustion method using an elemental analyzer (Euro EA3000, Eurovector, Italy). Total phenols were extracted by microwave‐assisted extraction and measured using the Folin–Ciocalteu method (Singleton et al. 1999). Neutral detergent solubles, hemicellulose, cellulose, lignin, and ash content were measured using neutral detergent fiber, acid detergent fiber, acid detergent lignin, and dry ashing methods (Straková et al. 2010). Neutral detergent solubles refer to cellular constituents dissolved during neutral detergent extraction and are preferentially utilized by microorganisms, with their concentration directly driving initial mass loss rates. The acid detergent method detects aromatic polymers resistant to acid hydrolysis, including both true lignin in vascular plants and *Sphagnum* lignin‐like phenolics. Although *Sphagnum* lacks true lignin but produces lignin‐like phenolic polymers, we quantified these compounds using the acid detergent method conventionally applied to lignin analysis (Straková et al. 2010). For consistency with methodological terminology, we hereafter refer to these *Sphagnum*‐specific compounds as “lignin‐like substances”.

### 2.3 Data Analysis

The percentage weight loss of plant litter was calculated using the formula:

$$D\left(\%\right)=100\times \left(W_{0}-W_{t}\right)/W_{0}$$

where *t* represents the decomposition time, *W*_0_ and *W*_*t*_ denote the dry weights of plant litter before and after decomposition, respectively, and *D* is the percentage weight loss of plant litter at time *t* (Lu et al. 2017).

The content change percentage of each chemical property in plant litter was calculated as:

$$\mathrm{PC}\left(\%\right)=100\times \left(C_{t}-C_{0}\right)/C_{0}$$

where *C*_0_ and *C*_*t*_ represent the chemical composition proportion in plant litter before and after decomposition, respectively, and *PC* represents the content change percentage of each chemical property at time *t*, relative to its initial content.

The long‐term decomposition trend of plant litter was modeled using the mass loss formula proposed by Harmon et al. (2009):

$$M_{t}=M_{0}e^{-\mathrm{kt}}+S$$

where *M*_*t*_ is the percentage of dry weight remaining, *t* is the decomposition time (years), *k* is the decomposition constant, and *S* is the asymptotic limit of litter decomposition. Decomposition trend fitting was performed using the improved one‐phase exponential decay model in GraphPad Prism 9.

The half‐life of plant litter decomposition (*t*_0.5_), defined as the time required for 50% of the initial mass to decay, was calculated as:

$$t_{0.5}=0.6931/k$$

Two‐way ANOVA was used to evaluate the effects of plant functional group and geographic origin on initial chemical components, initial stoichiometric ratios, weight loss percentage, and content change percentage of chemical properties. One‐way ANOVA was further applied to assess the effect of geographic origin on these indicators for the same genus plants. Duncan's method was used for multiple comparisons. *T*‐tests were used to determine whether the content change percentage of chemical properties showed a statistically significant difference from zero. Data analyses were conducted using SPSS 19.0, with a significance level of *α* = 0.05.

All analyses below were performed using R 4.4 (R Core Team 2024). To assess correlations between initial chemical properties and decomposition, Pearson correlation analysis was performed using the “stats” package. Principal component analysis (PCA) of initial chemical properties was conducted with the “ade4” package. We applied a multiple regression model to assess the influence of initial chemical qualities on litter decomposition (García‐Palacios et al. 2018). Based on the full model, potential subsets were ranked according to Akaike Information Criterion threshold (AICc) values to select the optimal subset, which was executed using the dredge function in the “MuMIn” package (Bartoń 2024). Before PCA and regression fitting, all initial chemical properties underwent SD standardization. The contribution of PCA components to litter decomposition was also evaluated.

## 3 Results

### 3.1 Initial Chemical Components

**FIGURE 1:**
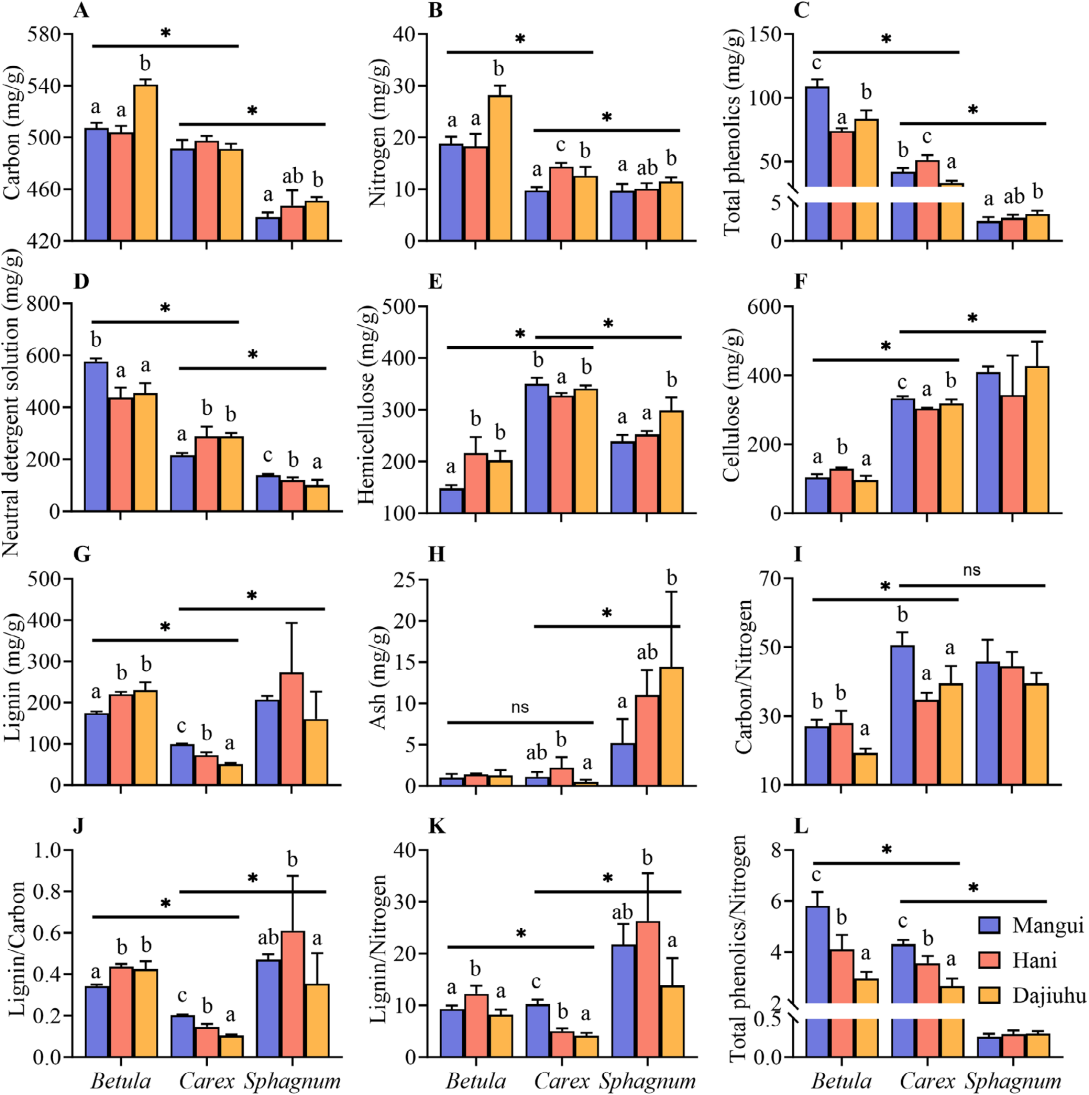
Effect of genus and origin of plants on initial chemical components and stoichiometric ratios of litters (mean + SEM, *n* = 5). Asterisks (*) indicate *p* < 0.05, and ns means no significant difference among genus. Bars with different letters indicate significant differences (*p* < 0.05), analyzed by Duncan tests. Lignin refers to lignin in vascular plants or lignin‐like substances in *Sphagnum*.

Initial chemical components and stoichiometric ratios of litter exhibited significant divergence across plant functional groups (*p* < 0.05, Table 2). Specifically, the concentrations of carbon (C), nitrogen (N), total phenolics (TPs), neutral detergent solubles (NDS), and the TPs/N (total phenolics to nitrogen) ratio in plant litter followed the order: *Betula* >*Carex* > *Sphagnum*. In contrast, the content of cellulose, ash, and the C/N ratio showed the opposite trend (Figure 1). The lignin‐like substances in *Sphagnum* and their ratios to carbon and nitrogen (lignin‐like/C and lignin‐like/N ratios) were significantly higher than lignin and its corresponding ratios (lignin/C and lignin/N ratios) in vascular plants. Among vascular plants, lignin content and its ratios to carbon and nitrogen were markedly higher in *Betula* than in *Carex*. The initial chemical components and stoichiometric ratios of plant litters were significantly influenced by geographic origin, except for cellulose, lignin/lignin‐like substances and ash content (*p* < 0.05, Table 2). Several key chemical indicators varied with latitudinal gradient (Figure 1): with latitude decrease, C, N, and hemicellulose content increased, whereas TPs, NDS, C/N ratio, and TPs/N ratio decreased.

**TABLE 2:** Two‐way analysis of variance for the effect of genus and origin of plants on initial chemical components of litters.

Index	Origin		Genus		Origin × Genus	
	*F*	*p*	*F*	*p*	*F*	*p*
C	**28.74**	**< 0.001**	**599.3**	**< 0.001**	**21.24**	**< 0.001**
N	**41.18**	**< 0.001**	**268.3**	**< 0.001**	**24.69**	**< 0.001**
TPs	**42.69**	**< 0.001**	**2344**	**< 0.001**	**65.02**	**< 0.001**
NDS	**7.397**	**0.002**	**903.3**	**< 0.001**	**30.48**	**< 0.001**
HCel	**17.93**	**< 0.001**	**327.8**	**< 0.001**	**14.52**	**< 0.001**
Cel	1.204	0.312	**152.5**	**< 0.001**	2.339	0.074
Lig	3.219	0.052	**43.61**	**< 0.001**	**3.940**	**0.009**
Ash	3.232	0.051	**35.19**	**< 0.001**	**3.292**	**0.021**
C/N	**19.13**	**< 0.001**	**110.0**	**< 0.001**	**8.046**	**< 0.001**
Lig/C	**3.867**	**0.030**	**42.36**	**< 0.001**	**3.217**	**0.023**
TPs/N	**84.50**	**< 0.001**	**692.5**	**< 0.001**	**27.45**	**< 0.001**
Lig/N	**10.05**	**< 0.001**	**55.27**	**< 0.001**	**4.218**	**0.007**

*Note:* Values in bold denote statistical significance (*p* < 0.05).

Abbreviations: Ash, ash content; C, carbon; Cel, cellulose; HCel, hemicellulose; Lig, lignin in vascular plants or lignin‐like substances in *Sphagnum*; N, nitrogen; NDS, neutral detergent solubles; TPs, total phenolics.

Except for cellulose content, a significant interaction between species and geographic origin was observed for the initial chemical properties and stoichiometric ratios of peatland litter (*p* < 0.05, Table 2). Notably, several key chemical properties showed opposite response patterns along the latitudinal gradient between *Betula* and *Carex* litter, such as TPs, NDS, cellulose, lignin (or lignin‐like substances) and lignin/C (or lignin‐like/C) ratio (Figure 1).

### 3.2 Functional Group Effects on Litter Decomposition

**FIGURE 2:**
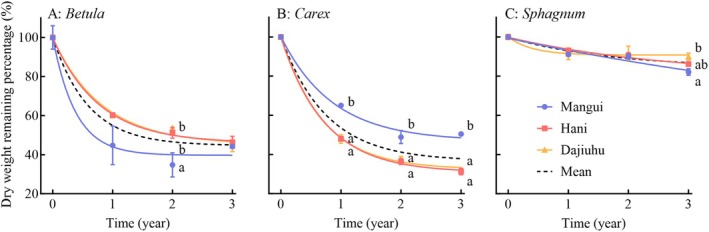
Differences in dry weight remaining percentage of plants litter from different origins over time in three functional groups of plants (mean ± SEM, *n* = 15). The fitting formula of the exponential decay with time for plants from different origins is presented in Table 4. Bars with different letters indicate significant differences (*p* < 0.05), analyzed by Duncan tests.

During the decomposition process, the decomposition percentages of plant litter from different functional groups showed highly significant differences (*p* < 0.001, Table 3, Figure 2). After 1 year, the average decomposition percentages of *Betula* and *Carex* were 45.0% and 46.3%, respectively, both exceeding that of *Sphagnum* (8.2%). After 2 and 3 years, decomposition rates across the three functional groups exhibited a clear hierarchical pattern from high to low: *Carex* > *Betula* > *Sphagnum*.

**TABLE 3:** Two‐way analysis of variance for the effect of genus and origin of plants on dry weight loss of litters.

Year	Origin		Genus		Origin × Genus	
	*F*	*p*	*F*	*P*	*F*	*p*
1	0.030	0.971	**110.3**	**< 0.001**	**6.904**	**< 0.001**
2	0.876	0.425	**257.73**	**< 0.001**	**8.364**	**< 0.001**
3	**4.676**	**0.016**	**611.9**	**< 0.001**	**17.93**	**< 0.001**

*Note:* Values in bold denote statistical significance (*p* < 0.05).

According to the fitting models derived from long‐term patterns of mass loss, *Sphagnum* showed the lowest mass loss (Table 4), indicating the greatest resistance to decomposition among the three functional groups. Based on the estimated *S* values from the model, the final mass retention for *Sphagnum* was 83.8%, much higher than that for *Betula* (44.7%) and *Carex* (36.7%). The half‐life of *Sphagnum* decomposition was 1.25 years, longer than that of *Betula* (0.41 years) and *Carex* (0.53 years).

**TABLE 4:** The parameters obtained by fitting formula of the exponential decay with time of litter decomposition ($M_{t}=M_{0}e^{-\mathrm{kt}}+S$) for different functional groups of plants from different origins.

Genus	Origin	*k*	*S*	Half‐life	*R* ^2^
*Betula*	Mangui	2.62	39.7	0.26	0.78
	Hani	1.30	46.0	0.53	0.96
	Dajiuhu	1.21	45.3	0.57	0.97
	Mean	1.68	44.7	0.41	0.28
*Carex*	Mangui	1.16	46.8	0.60	0.96
	Hani	1.37	30.8	0.51	0.99
	Dajiuhu	1.44	32.4	0.48	0.99
	Mean	1.32	36.7	0.53	0.44
*Sphagnum*	Mangui	0.13	47.5	5.46	0.82
	Hani	0.36	79.7	1.93	0.81
	Dajiuhu	2.49	90.8	0.28	0.37
	Mean	0.55	83.8	1.25	0.10

**FIGURE 3:**
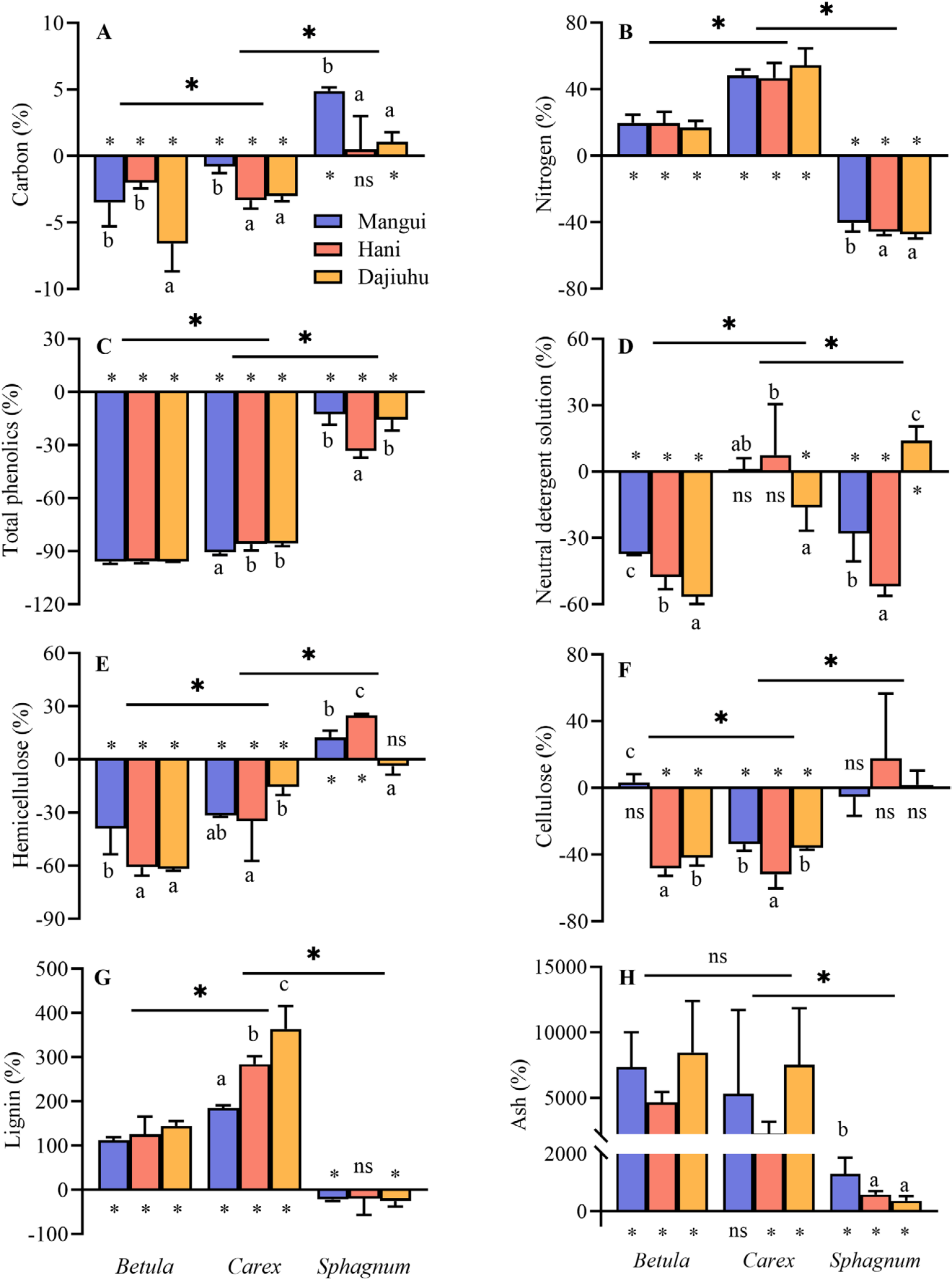
Effect of genus and origins of plants on the change percentage in the chemical composition content of litter during decomposition (mean + SEM, *n* = 5). Asterisks denote a significant difference among genus or whether the percentage change in chemical composition was a significant difference from zero. Asterisks (*) indicate *p* < 0.05, and ns means no significant difference. Bars with different letters indicate significant differences (*p* < 0.05), analyzed by Duncan tests. Lignin refers to lignin in vascular plants or lignin‐like substances in *Sphagnum*.

The chemical composition of plant litter overall changed significantly after 3 years of decomposition compared to the initial state, except for N (*p* < 0.01, Table 5). The contents of C, TPs, NDS, hemicellulose, and cellulose all decreased, while lignin/lignin‐like substances and ash contents increased (Figure 3). Further analysis revealed significant differences in chemical composition change among the three functional groups (*p* < 0.001, Table 6), with *Sphagnum* showing an opposite trends to vascular plants in C, N, hemicellulose, cellulose, and lignin/lignin‐like substances contents (Figure 3).

**TABLE 5:** *T*‐test for the significance of percentage variations in change percentage of chemical properties of plant litter between pre‐ and post‐decomposition samples.

Index	Number	Mean value	Standard error	*t*	*p*
C	45	−1.425	0.5025	−2.836	0.007
N	45	8.001	5.982	1.338	0.188
TPs	45	−67.88	5.167	−13.14	< 0.001
NDS	45	−23.92	4.035	−5.928	< 0.001
HCel	45	−23.33	4.496	−5.188	< 0.001
Cel	45	−21.61	4.160	−5.193	< 0.001
ADL	45	127.6	19.91	6.409	< 0.001
Ash	45	4192	610.4	6.867	< 0.001

Abbreviations: Ash, ash content; C, carbon; Cel, cellulose; HCel, hemicellulose; Lig, lignin in vascular plants or lignin‐like substances in *Sphagnum*; N, nitrogen; NDS, neutral detergent solubles; TPs, total phenolics.

**TABLE 6:** Two‐way analysis of variance for the effect of genus and origin of plants on the change rate in the chemical composition content of litter during decomposition.

Index	Origin		Genus		Origin × Genus	
	*F*	*p*	*F*	*p*	*F*	*p*
C	20.67	< 0.001	89.82	< 0.001	8.861	< 0.001
N	0.567	0.572	955.9	< 0.001	1.895	0.133
TPs	12.68	< 0.001	2086	< 0.001	20.64	< 0.001
NDS	5.194	0.010	73.01	< 0.001	30.32	< 0.001
HCel	2.482	0.098	181.4	< 0.001	12.26	< 0.001
Cel	5.166	0.011	39.86	< 0.001	9.823	< 0.001
Lig	25.20	< 0.001	481.4	< 0.001	16.68	< 0.001
Ash	3.729	0.034	15.61	< 0.001	1.086	0.378

Abbreviations: Ash, ash content; C, carbon; Cel, cellulose; HCel, hemicellulose; Lig, lignin in vascular plants or lignin‐like substances in *Sphagnum*; N, nitrogen; NDS, neutral detergent solubles; TPs, total phenolics.

### 3.3 Geographic Origin Effects on Litter Decomposition

Overall, peatland plant litter from different geographic origins exhibited significant differences in decomposition after 3 years (*p* < 0.05, Table 3). The average decomposition percentage of litter from Mangui peatland (41.1%) was lower than that from Hani (45.4%) and Dajiuhu peatlands (44.4%). However, the influence of geographic origin varied among the three functional groups (Figure 2, Table 3). *Carex* litter from Mangui showed the least decomposition, but both *Betula* and *Sphagnum* litters from Mangui showed the highest decomposition percentages.

Decomposition model analysis demonstrated that geographic origin affected decomposition (Table 4). *Betula* litter from the Mangui peatland was predicted to retain 39.7% of its initial dry weight, less than that from the Hani (46.0%) and Dajiuhu peatlands (45.3%), with corresponding estimated half‐ life of 0.26, 0.53, and 0.57 years, respectively. In contrast, for *Carex*, the final dry weight remaining percentage from Mangui was 46.8%, higher than that from Hani (30.8%) and Dajiuhu (32.4%) with corresponding estimated half‐lives of 0.60, 0.51, and 0.48 years, respectively. For *Sphagnum*, the final mass retention increased with decreasing latitude.

During the decomposition process, changes in the chemical composition (excluding nitrogen and hemicellulose) of litter from different origins showed significant differences (*p* < 0.05, Table 6, Figure 3). Lignin/lignin‐like substances content increased after decomposition, with greater accumulation at lower latitudes. TPs, NDS, and cellulose contents generally decreased, showing maximal reduction in litter from Hani peatland. Additionally, there was a significant interaction between functional group and geographic origin on the rate of change for most chemical components, excluding nitrogen and ash (*p* < 0.001, Table 6).

### 3.4 Relationship Between Initial Chemical Composition and Decomposition

**FIGURE 4:**
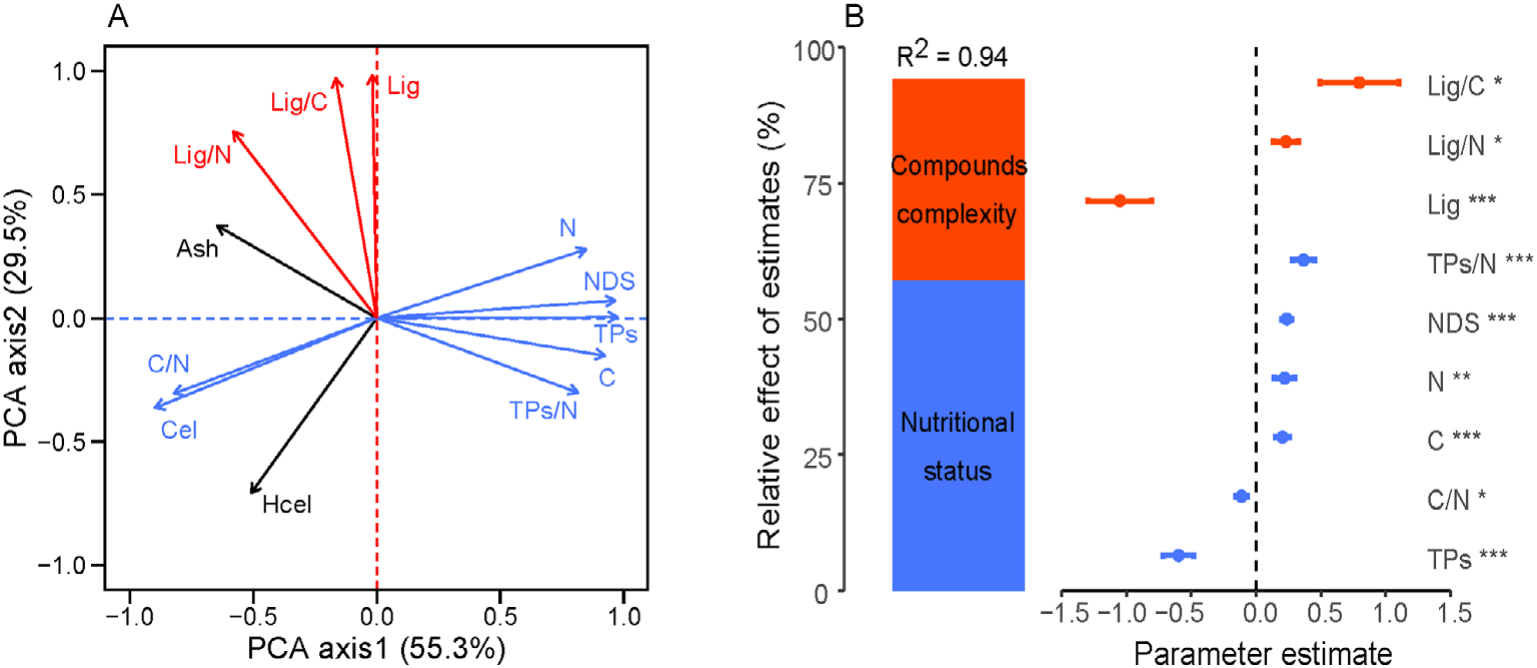
Relationships between the initial chemical compositions and decomposition rates over 3 years in peatland plant litters. (A) Principal Component Analysis (PCA) of the initial chemical compositions in peatland plant litters, displaying the first two principal components. (B) Relative influence of various predictors on litter decomposition rates. The plot shows standardized regression coefficients (with 95% confidence intervals) for each predictor in the model, alongside the percentage of variance explained by each PCA axis. Blue bars indicate indicators of litter compound complexity and composition, while red bars represent indicators of litter nutritional status and composition. Asterisks signify the statistical significance of initial biochemical properties on decomposition rates, with *p < 0.05, **p < 0.01, and ***p < 0.001. Lignin refers to lignin in vascular plants or lignin‐like substances in *Sphagnum*.

Pearson correlation analysis revealed that plant decomposition percentages in peatlands were significantly correlated with the initial biochemical properties of plant litter, except for hemicellulose (*p* < 0.01, Table 7). Principal component analysis (PCA) of the initial biochemical properties showed that the first two principal components jointly explained 84.8% of the variation in biochemical composition (Figure 4A). The first principal component axis was strongly positively correlated with C, N, TPs, NDS, and TPs/N ratio and negatively correlated with cellulose and the C/N ratio. The first axis reflected the nutritional status of the litter. The second principal component axis showed a strong positive correlation with lignin/lignin‐like substances content, lignin/C ratio (or lignin‐like/C ratio), and lignin/N ratio (or lignin‐like/N ratio), representing the complexity of the litter compounds.

**TABLE 7:** The relationship between mass loss of peatland plant litter after different years of decomposition and the chemical traits of litter combinations at different stages.

Index	Dry weight loss		
	1 year	2 years	3 years
C	0.740**	0.761**	0.794**
N	0.428**	0.394**	0.448**
TPs	0.752**	0.753**	0.701**
NDS	0.734**	0.724**	0.687**
HCel	−0.043	0.015	0.078
Cel	−0.573**	−0.558**	−0.537**
Lig	−0.451**	−0.493**	−0.508**
Ash	−0.631**	−0.674**	−0.730**
C/N	−0.448**	−0.395**	−0.430**
Lig/C	−0.542**	−0.586**	−0.605**
TPs/N	0.831**	0.872**	0.787**
Lig/N	−0.726**	−0.749**	−0.781**

*Note:* Values in the table are correlation coefficients by Pearson's correlation analysis.

Abbreviations: Ash, ash content; C, carbon; Cel, cellulose; HCel, hemicellulose; Lig, lignin in vascular plants or lignin‐like substances in *Sphagnum*; N, nitrogen; NDS, neutral detergent solubles; TPs, total phenolics.

** *p* < 0.01.

According to AICc, the optimal model explaining litter decomposition included nutritional status indicators C, N, TPs, NDS, cellulose, C/N ratio, and TPs/N ratio, and compound complexity indicators lignin/lignin‐like substances, lignin/C ratio (or lignin‐like/C ratio) and lignin/N ratio (or lignin‐like/N ratio). Nutritional status and compound complexity indicators explained 60.8% and 39.1% of litter decomposition, respectively (Figure 4B). Among these, factors such as lignin/lignin‐like substances, lignin/C ratio (or lignin‐like/C ratio), TPs, and TPs/N ratio were the primary contributors to litter decomposition. The forest plot derived from multiple regression analysis (Figure 4B) demonstrated that TPs and lignin (including *Sphagnum*‐specific lignin‐like substances) significantly inhibited decomposition, with standardized regression coefficients of *β* = −0.62 (*p* < 0.001) and *β* = −0.93 (*p* < 0.001). In contrast, the TPs/N ratio and lignin/C ratio exhibited significant facilitatory effects (*β* = 0.36, *p* < 0.001; *β* = 0.58, *p* < 0.05, respectively).

## 4 Discussion

### 4.1 Functional Group Dependence of Litter Decomposition in Peatlands

Species identity is a key driver of variation in plant litter decomposition (Cornwell et al. 2008; Erdenebileg et al. 2023). Different plant functional groups have evolved specific traits, structures, and morphologies during long‐term environmental adaptation, resulting in variations in the concentration of soluble components in their litters (Yu et al. 2024). The hierarchical decomposition patterns among functional groups—graminoids (*Carex*) > shrubs (*Betula*) > mosses (*Sphagnum*)—were driven by divergent biochemical traits (Moore and Basiliko 2006). *Sphagnum* litter exhibited the highest recalcitrance, attributable to its high lignin‐like phenolic polymers and low nitrogen content. These traits synergistically limit microbial access to labile carbon substrates, as lignin‐like compounds physically shield cellulose while phenolic metabolites inhibit extracellular enzyme activity (Bengtsson et al. 2018; Fenner and Freeman 2020). In contrast, *Carex* litter decomposed rapidly, aligning with its low lignin/N ratio and high hemicellulose content, which enhance microbial utilization efficiency (Straková et al. 2010).

There is a clear hierarchical structure in the decomposability of peatland plant litters, indicating significant functional group dependence. This result supports our first hypothesis and aligns with the findings of Moore and Basiliko (2006), who reported that litter decomposition rates in peatlands follow a sequence from fastest to slowest: sedge leaves > shrub leaves > deciduous tree leaves > coniferous tree leaves > *Sphagnum* fragments > woods. However, this hierarchical pattern only became apparent in the third year of decomposition, with model projections suggesting its persistence over time. Compared to forests, the delayed manifestation in peatlands likely stems from slow decomposition (Wang et al. 2021). These findings align with Moore and Basiliko (2006), who documented similar lag phases in Canadian boreal peatlands but observed extended delays, potentially attributable to warmer mean annual temperatures at Hani peatland relative to the boreal peatland.

### 4.2 Influence of Geographic Origin on Litter Decomposability

Our study revealed significant impacts of geographic origin on plant litter decomposition rates. Litter from the northernmost Mangui peatland exhibited the highest decomposition resistance, partially validating our second hypothesis. Climate regulates litter decomposability by modifying plant traits (Fortunel et al. 2009). Cold, nutrient‐poor environments drive plants to produce litter with low nitrogen content, elevated carbon to nitrogen ratios, and high total phenolic content, which collectively constrain microbial decomposition (Wu et al. 2025). Conversely, lower latitudes may promote the production of more labile litter in peatlands.

However, geographic effects on decomposability are mediated by both dominant microbial communities and decomposition‐site conditions (Hoyos‐Santillan et al. 2018; Joly et al. 2023). Notably, Litter from Hani and Dajiuhu peatlands did not show differences in decomposition percentages when decomposed in Hani peatland, regardless of plant species. Typically, differences in the biochemical traits of litter caused by geographic origin could determine decomposition variations. However, although the geographical origin led to statistically significant differences in certain biochemical traits between litter from Hani and Dajiuhu peatlands, the magnitude of these differences was relatively small. Consequently, other factors, such as the adapted decomposer community or local environmental conditions at the Hani site, likely overrode the influence of these subtle biochemical variations. This phenomenon aligns with the concept of “home field advantage” in plant litter decomposition, which states that specialized decomposers efficiently break down litter in its native environment (Palozzi and Lindo 2017). For example, decomposition of autochthonous litter was maximized due to microbial community adaptation, irrespective of the nutrient status of the site (Hoyos‐Santillan et al. 2018). Therefore, studying the influence of geographic origin on intrinsic litter decomposability requires approaches that exclude the confounding effect of the home field advantage.

A significant interaction effect was observed between geographic origin and plant functional groups on litter decomposition. The effect of geographic origin on the decomposition of *Carex* differed from that of the other two functional groups, supporting Hypothesis 3. The specific decomposability of plant litter in response to environmental changes is related to plant functional groups. As herbivory pressure on woody plants increases with decreasing latitude, plant defense such as increased leaf lignin content also intensifies (Wang et al. 2016). In this study, the lignin content of *Betula* leaves increased with decreasing latitude, which may result in high decomposition resistance in Hani and Dajiuhu peatlands. Most graminoids, such as *Carex*, are ruderal type species whose growth rates increase under improved environmental conditions, such as climate warming (Yao et al. 2022), resulting in more easily decomposable litter (Garnier et al. 2004). We observed that the decomposition rate of *Sphagnum* diminished progressively as the latitude of its geographic origin decreased, while the percentage of final dry weight remaining increased. This phenomenon may be attributed to rising temperatures inducing competition from vascular plants and drought, which inhibited the growth of slow‐growing *Sphagnum* (Norby et al. 2023), resulting in more decay resistance in litter. Therefore, different plant functional groups exhibit distinct trade‐offs between nutrient acquisition and defense response to changing latitude, leading to variations in leaf decomposability (Kazakou et al. 2019).

### 4.3 Initial Chemical Components Influencing Litter Decomposition

Our study found that decomposition percentages in peatlands were significantly correlated with the initial biochemical properties, except hemicellulose, of plant litters. Principal component analysis (PCA) revealed that the biochemical properties influencing litter decomposition could be categorized into two groups: nutritional status and compound complexity. The initial nutritional status properties explained 60.8% of the variance in litter decomposition, with TPs and its ratio to N being key determinants of decomposition. The latitudinal decline in TPs/N ratios mechanistically explains the enhanced decomposability at lower latitudes. In peatlands, TPs inhibit microbial activity, thereby reducing decomposition rates (Bragazza et al. 2009; Fenner and Freeman 2020). Peatland litter contains low concentrations of nutrients such as N and P, resulting in slow decomposition rates (Limpens and Berendse 2003; Moore and Basiliko 2006). Increasing nutrient levels in litters can promote decomposition (Shah et al. 2024). Song et al. (2011) demonstrated that increasing nitrogen levels improved peatland litter quality, enhanced microbial activity, and accelerated the decomposition rate of *Calamagrostis angustifolia*. Therefore, the initial nutrient availability is a critical factor affecting decomposition.

Additionally, lignin content was a major factor in determining compound complexity in this study. Lignin is a water‐insoluble macromolecule with a complex chemical structure that decomposes slowly (Berg and Mcclaugherty 2020). The lignin‐like substance in *Sphagnum* and canonical lignin in vascular plants serve analogous functional roles in plant tissues, such as providing mechanical reinforcement to cell walls and shielding the polysaccharides against decomposers (Bengtsson et al. 2018). The higher lignin‐like substance content in *Sphagnum* (vs. vascular plant lignin) may reflect convergent evolution of decay‐resistant polymers in waterlogged environments. Lignin/or lignin‐like substance content is often negatively correlated with litter decomposition rate, which is consistent with the inhibitory effect of lignin/lignin‐like substance on plant litter decomposition in this study. Therefore, the biochemical properties of plant litter play a crucial role in controlling decomposition rates (Moore and Basiliko 2006; Zheng et al. 2017).

The legacy effects of leaf biochemical properties and structural characteristics profoundly affect litter quality (Liu et al. 2018; Kazakou et al. 2019). For example, N content in forest litter across Eurasia increases with annual mean temperature (Liu et al. 2006). In this study, the initial chemical qualities of litter were influenced by both geographic origin and functional group of the plants. Specifically, the contents of C and N in plant litter followed the order: *Betula*>*Carex*>*Sphagnum*, whereas the C/N ratio exhibited an opposite rank trend. C and N contents increased with decreasing latitude, whereas the C/N ratio decreased with decreasing latitude. Thus, these findings suggest that climate change can indirectly regulate litter decomposition by altering litter quality (Liu, Zhang, et al. 2024) and climate warming may prompt peatland vegetation to produce more easily decomposable litters.

The experimental use of air‐dried fresh leaves as substitutes for naturally senesced litter in this study provided methodological advantages by reducing pre‐decomposition variability and minimizing microbial pre‐colonization heterogeneity, thereby effectively revealing latitudinal gradients' impacts on litter decomposability. However, it should be noted that nutrient resorption during leaf senescence may lead to amplified initial decomposition rates and potential alterations in interspecific differences when using fresh leaves (Guo et al. 2024). Therefore, caution should be exercised when extrapolating leaf decomposition based on fresh leaf trait data. We recommend future studies employ the method of combining fresh leaves with naturally senesced litter to obtain a more comprehensive understanding of litter decomposition processes.

### 4.4 Continuous Carbon Accumulation in Peatlands

The long‐term dynamics of mass loss assumes that a portion of litter decomposes at an extremely slow rate or remains undecomposed, adding a constant term *S* to the single exponential decay model (Harmon et al. 2009; Currie et al. 2010). During decomposition, the chemical composition of the litter changes significantly. The contents of chemical components that are difficult to decompose, such as ash and lignin/lignin‐like substances, show an increasing trend, resulting in the decomposition rate approaching zero and the final dry weight remaining percentage approaching *S*. By applying the formula for the long‐term dynamics of mass loss to calculate the limit:

$$M=\lim_{t\to \infty }\frac{\sum_{0}^{t}\left(M_{0}e^{-\mathrm{kt}}+S\;\right)}{t}=S$$

It can be inferred that the constant *S* approximates the long‐term net accumulation rate of plant litter during the long‐term balance between production and decomposition. In this study, *S* was greater than zero, indicating that plant litter production exceeded decomposition in the three peatlands in the long term, resulting in a net accumulation of organic matter. A substantial volume of peat remains isolated from the atmosphere due to waterlogging, thereby forming a persistent carbon stock. Despite site differences, all three peatlands are expected to maintain carbon accumulation due to environmental constraints and litter quality limiting decomposition rates (Ofiti et al. 2023; Tolunay et al. 2024).

### 4.5 Impact of Climate Warming on Carbon Accumulation in Peatlands

In this study, irrespective of plant functional group, warming associated with lower latitudes may increase the production of more easily decomposable litter in peatlands, potentially accelerating litter decomposition. Climate warming and nitrogen deposition are transforming cold, wet, and nutrient‐poor conditions of peatlands, leading to an increased dominance of vascular plants in many northern peatlands (Wang et al. 2015; Gong et al. 2024). In this study, the decomposition rates of vascular plants, such as *Betula* and *Carex*, were greater than those of *Sphagnum*, suggesting that shrub/graminoid expansion could accelerate ecosystem‐level decomposition. In peatlands, carbon accumulation is primarily due to low decomposition rates (Liu, Han, and Wang 2024). Consequently, climate change is likely to ultimately diminish the carbon accumulation function of peatlands. However, we must admit that our predictions are based on experiments using single‐species litters. Plant litters in peatlands often are mixed and decompose simultaneously, and their decomposition can be affected by plant diversity and composition (Zhou et al. 2020).

## 5 Conclusion

Our study demonstrated that the decomposability of plant litter decreased with increased latitude, primarily driven by its biochemical legacy effect. This pattern resulted from systematically lower total phenolic to nitrogen (TPs/N) ratios in southern‐origin litter. Decomposition patterns and environmental responses exhibited strong functional group specificity, governed by initial biochemical properties. This was particularly evident for vascular plants like *Carex* spp., whose expansion in warming high‐latitude peatlands may accelerate litter decomposition. Long‐term mass loss modeling projections suggested that these peatlands could maintain net carbon accumulation under current climate scenarios. However, projections of future carbon dynamics remain uncertain, given the uncertainties associated with climate‐driven shifts in plant functional composition and decomposition processes. Future investigations should prioritize three critical areas: long‐term decomposition monitoring, microbial community dynamics, and experimental warming studies.

## Author Contributions

**Jinze Ma:** data curation (lead), formal analysis (lead), investigation (lead), methodology (supporting), visualization (lead), writing – original draft (lead). **Yong‐Da Chen:** software (lead), writing – review and editing (supporting). **Si‐Nan Wang:** investigation (supporting), validation (lead). **Jing Zeng:** investigation (supporting). **Chao Liu:** methodology (supporting). **Zhao‐Jun Bu:** conceptualization (lead), funding acquisition (lead), methodology (lead), project administration (lead), resources (lead), supervision (lead), writing – review and editing (lead).

## Conflicts of Interest

The authors declare no conflicts of interest.

## Supporting information


Appendix S1.

## Data Availability

The data that support the findings of this study are available in the Supporting Information of this article.
